# Comparative Metabolite Profiling of Different Solvent Extracts of *Argemone ochroleuca* Sweet and *Argemone mexicana* Linn Shoots and Roots Using Liquid Chromatography–Mass Spectrometry-Based Metabolomics and Molecular Networking

**DOI:** 10.3390/molecules31152734

**Published:** 2026-08-06

**Authors:** Nezelo Trizer Mlombo, Fikile Nelly Makhubu, Zakheleni Palane Dube, Ntakadzeni Edwin Madala, Thilivhali Emmanuel Tshikalange

**Affiliations:** 1 Department of Plant and Soil Sciences, University of Pretoria, Hatfield, Pretoria 0028, South Africa; 2 Department of Botany and Plant Biotechnology, University of Johannesburg, Johannesburg 2092, South Africa; fmakhubu@uj.ac.za; 3 School of Biology and Environmental Sciences, University of Mpumalanga, Mbombela 1200, South Africa; zakheleni.dube@ump.ac.za; 4 Centre of Excellence in Mass Spectrometry for Southern Africa (SEMSSA), Faculty of Science, Engineering and Agriculture, University of Venda, Thohoyandou 0950, South Africa; ntakadzeni.madala@univen.ac.za; 5 Department of Life and Consumer Sciences, University of South Africa, Florida, Johannesburg 1709, South Africa; tshikte@unisa.ac.za

**Keywords:** *Argemone*, LC-MS, metabolites, metabolomics, molecular network

## Abstract

*Argemone ochroleuca* and *Argemone mexicana* are widespread weed species known for being rich in various secondary metabolites. However, a comprehensive understanding of their chemical diversity remains limited. Insufficient information exists on metabolite variation between plant parts and the influence of solvent polarity on metabolite recovery. Therefore, this study aimed to characterize the metabolomic profiles of the shoots and root extracts of both species using solvents of varying polarity to evaluate plant part-specific metabolite distribution and solvent effects on metabolome coverage. Untargeted ultra-high-performance liquid chromatography–quadrupole time-of-flight mass spectrometry (UPLC-QTOF-MS)-based metabolomics and molecular networking were employed for metabolite analysis. Principal component analysis (PCA) did not reveal clear clustering of *A. ochroleuca* and *A. mexicana*, suggesting similarities in their metabolomic profiles. A total of 15 and 13 metabolite classes, yielding 59 and 54 metabolites, were identified in *A. ochroleuca* and *A. mexicana*, respectively, including flavonoids, terpenoids, phenolic compounds, fatty acids, and monoterpenoids. *Argemone ochroleuca* exhibited higher metabolite abundance, particularly in methanol and acetone extracts, and with shoots showing higher abundance than roots, with flavonoids being the dominant class. The findings show that LC-MS metabolomics, molecular networking, and careful solvent selection are effective for identifying key metabolites with potential applications in crop protection.

## 1. Introduction

*Argemone ochroleuca* and *Argemone mexicana* are two major weed species of the Papaveraceae family. These two species have been reported to have a variety of chemical compounds with biologically active properties [1,2,3]. As reported by Reyes-Luna et al. [4] and Vardeman et al. [5], metabolomic profiling on *A. ochroleuca* and *A. mexicana* extracts revealed that the extracts are rich in alkaloids such as berberine, allocryptopine and protopine. Other studies have also identified several secondary metabolites, including oxyberberine; 1,3-bis (3-phenoxyphenoxy)-; atropine; argemexicin; argemonin; pentanoic acid; and pentanoic acid, 5-hydroxy-, 2,4-di-t-butylphenyl esters [6,7,8,9]. Although extensive research has been conducted to characterize the compounds in these two plants using GC-MS or isolation methods [1,4,9,10] and to evaluate their biological activities, many of the specific phytochemicals responsible for these effects have not yet been thoroughly elucidated [11].

The limited literature which exists on metabolite extraction in *A. ochroleuca* and *A. mexicana* uses different solvent systems and other methods like LC-MS to assess their metabolomic profiles. Most studies rely on a single extraction solvent, which may not capture the full chemical diversity of these plants [10,12]. The effect of solvent polarity on the extraction of metabolites from plant materials therefore remains inadequately explored. In order to improve metabolomic analyses and optimize extraction procedures, it is critical to address this gap. Since bioactive compounds differ in polarity and solubility, the choice of solvent directly affects the range of metabolites extracted [13,14]. Untargeted metabolomics allows for the characterization, quantification, and optimization of active metabolites of plant crude extracts using various solvents with different polarities, without the need for isolation techniques, allowing for a thorough examination of a complex mixture of molecules with potential use in biological activity tests and assays [14].

The plant metabolome is essentially complex due to the large number of structurally diverse metabolites that exhibit a broad spectrum of physicochemical characteristics [15]. The production and accumulation of metabolites are highly plant part-specific, driven by diverse gene expression, subcellular compartmentalization and other factors [16]. This complexity is a fundamental problem in metabolomics, because no single analytical or extraction approach can capture the whole metabolome [15,17,18]. Metabolomics research should use diverse solvent systems and tissue-specific extraction procedures to address selective detection [15].

The identification of metabolites is the most crucial and difficult process during the metabolomics pipeline [19]. Metabolomic profiling has been developed to be a strong analytical method, providing unparalleled insights into the intricate metabolic networks and biochemical contents of plant extracts [17,20]. However, the immense phytochemical variety inherent in the plant chemical space poses both an incredible opportunity and a difficult problem for scientists striving to harness these metabolites efficiently [17]. Even though molecular networking allows for the visualization of several classes of secondary metabolites in plants, compound annotation is currently restricted to the most widely researched molecules [19]. Currently, most work in chemical characterization is conducted using traditional methods of chemical identification, in which collected mass spectrometry (MS) signals are compared to those of previously known metabolites reported elsewhere in the literature [21]. This approach, however, has important limitations due to the limited information available for some uncharacterized metabolites [21]. However, molecular networking enables the grouping of unknown compounds into molecular families based on spectral similarity, allowing for deeper investigation and the potential discovery of novel metabolites [22]. These approaches can similarly be applied to the metabolomic profiling of plants such as *A. ochroleuca* and *A. mexicana*.

There are still more metabolites within *A. ochroleuca* and *A. mexicana* that have currently not been identified or reported in the existing literature due to the limitations of the current metabolomic profiling techniques, which leaves a huge gap in the chemical constituents of the plants. Therefore, the current study aimed to use LC-MS-based untargeted metabolomics to investigate the metabolomic profiles of extracts of *A. ochroleuca* and *A. mexicana* obtained using solvents of varying polarities: water, methanol, acetone and dichloromethane on the shoots and roots. This study will provide new scientific evidence for the potential use of *A. ochroleuca* and *A. mexicana* as crop protective agents, establishing the groundwork for future research into the active metabolites.

## 2. Results

**Figure 1:**
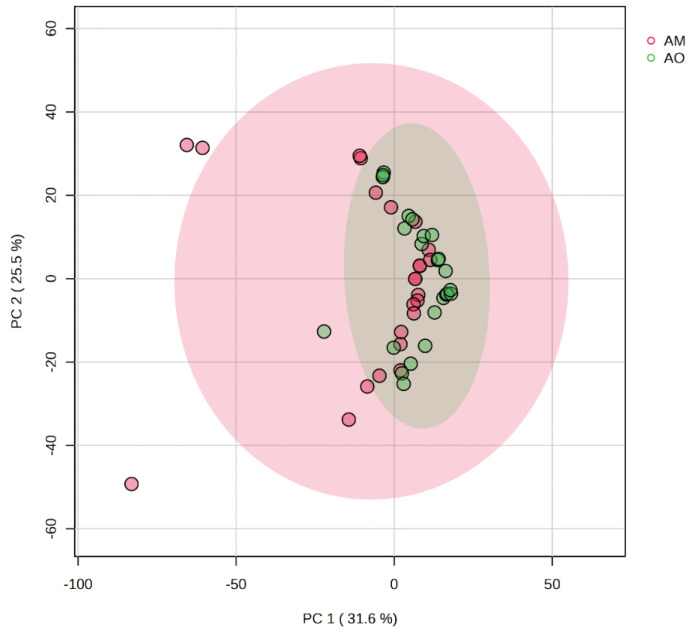
PCA score scatterplot of *Argemone ochroleuca* (AO) and *Argemone mexicana* (AM) extracts extracted with water, methanol, acetone and dichloromethane. The different samples are represented by different colors as shown in the legend above. Different sample groups are represented by the distinct colors: green—*Argemone ochroleuca* (AO)—and red—*Argemone mexicana* (AM). The generated two-component model explained 57.1% of the total variance.

A comprehensive LC-MS analysis was conducted on *A. ochroleuca* and *A. mexicana* shoot and root extracts. PCA was carried out to evaluate the overall metabolomic variation and clustering among the plant species and the extracts. The PCA score plot revealed no clear separation between the two plant species (Figure 1), as the sample groups (*A. mexicana* (AM) (red) and *A. ochroleuca* (AO) (green)) overlapped substantially. This could indicate similar shared metabolic profiles; however, some samples were observed to cluster separately demonstrating considerable underlying metabolic variations.

**Figure 2:**
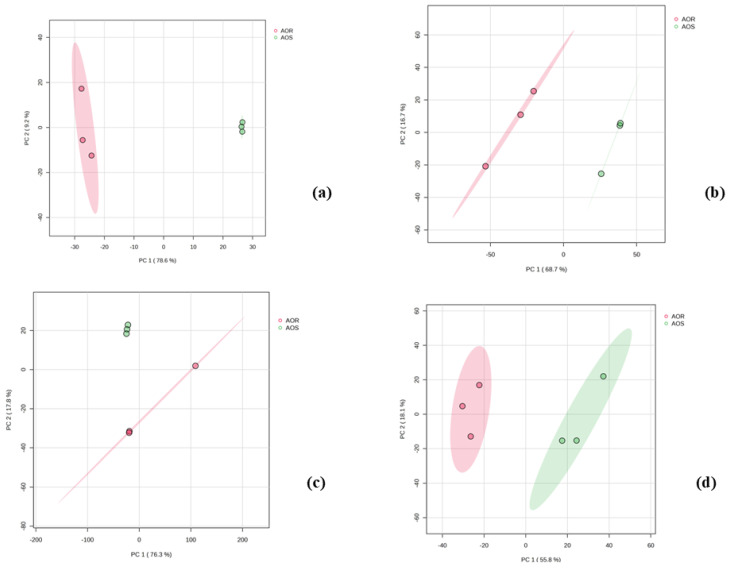
PCA score scatterplots of *Argemone ochroleuca* shoot and root (**a**) water, (**b**) methanol, (**c**) acetone and (**d**) dichloromethane extracts. The different samples are represented by different colors as shown in the legend above: green, *Argemone ochroleuca* shoot (AOS), and red, *Argemone ochroleuca* root (AOR).

**Figure 3:**
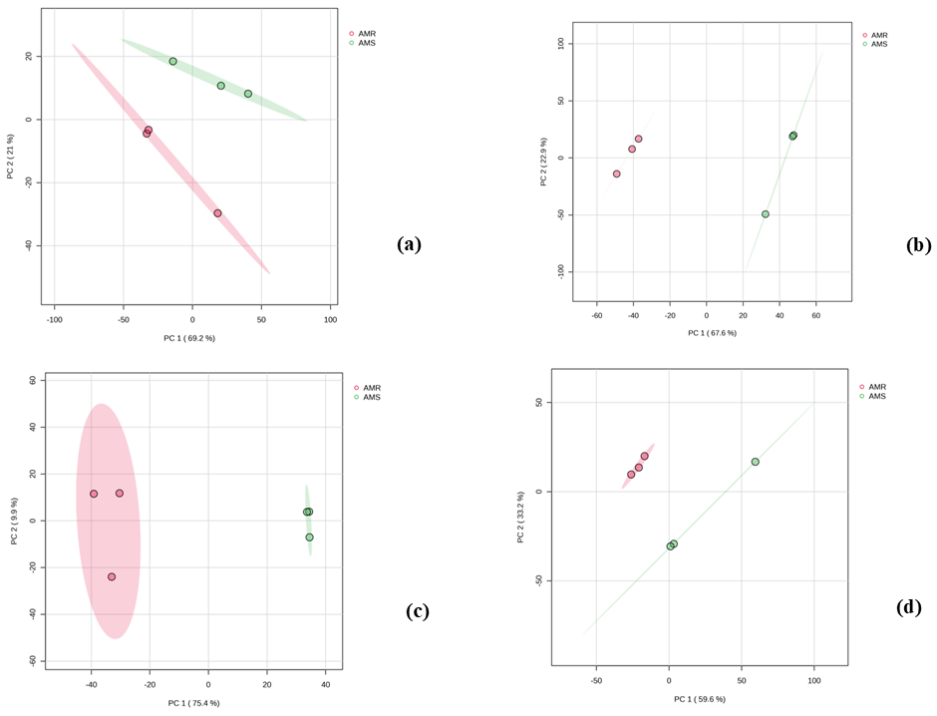
PCA score scatterplots of *Argemone mexicana* shoot and root (**a**) water, (**b**) methanol, (**c**) acetone and (**d**) dichloromethane extracts. The different samples are represented by different colors as shown in the legend above: green, *Argemone mexicana* shoot (AMS) and red, *Argemone mexicana* root (AMR).

As there was no clear separation between the plant species, PCA was performed separately to evaluate metabolomic profiles of shoot and root extracts of *A. ochroleuca* (Figure 2) and *A. mexicana* (Figure 3) prepared using different solvents (water, methanol, acetone and dichloromethane), to assess the similarities and differences in metabolite composition among the analyzed samples. The PCA score plot revealed distinct clustering of root and shoot extracts of *A. ochroleuca* (AOR and AOS). The generated models explained 87.8% of the variation for water (a), 85.4% for methanol (b), 94.1% for acetone (c) and 73.9% for dichloromethane (d). A similar pattern of distinct group separation for *A. mexicana* (AMR and AMS) was observed. The models explained 90.2% of the variance for water (a), 90.5% for methanol (b), 85.3% for acetone (c), and 92.8% for dichloromethane. The different grouping may be due to their metabolic profiles, showing variations in metabolite composition among *A. ochroleuca* and *A. mexicana* samples. Samples with comparable metabolic compositions grouped closely together, while samples with different metabolic profiles were split along the principal component axes.

**Figure 4:**
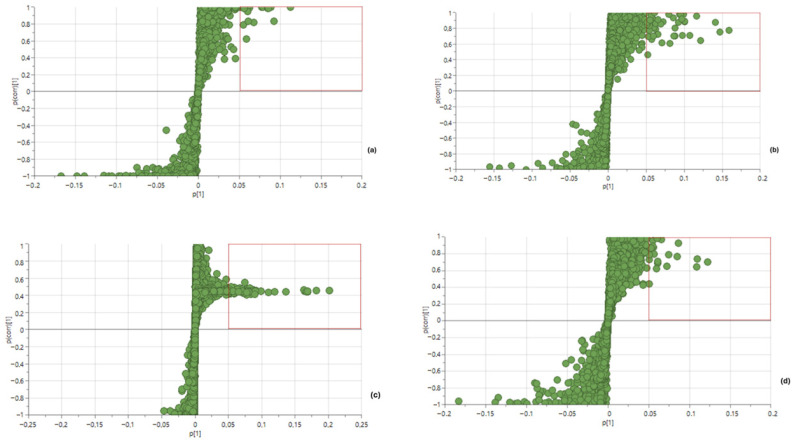
OPLS-DA-based S-plots showing differential distribution of key metabolic features (top right quadrant) in *Argemone ochroleuca* shoot and root water (**a**), methanol (**b**), acetone (**c**) and dichloromethane (**d**) extracts. Features located at the extremes of the plot (top right quadrant highlighted in red) represent key metabolic features with a strong contribution to the model.

The S-plots were used to identify the metabolic features that contributed most to the differentiation between the shoot and root extracts of *A. ochroleuca* obtained using water, methanol, acetone and dichloromethane extracts (Figure 4). Metabolic features with p[1] scores ≥ 0.05 in the S-plot were selected, leading to the annotation of 14 key metabolites associated with solvent type and plant part in *A. ochroleuca* extracts (Table 1): one from water, six from methanol, 12 from acetone, and eight from dichloromethane. Metabolites such as 6-gingerol; 4-Decylbenzenesulfonic acid; 5,6,2′-trimethoxyflavone; feruloyltyramine; 1-[2-(1,3-benzodioxol-5-yl)-3-methyl-1-benzofuran-5-yl]propane-1,2-diol; 9,12,13-trihydroxyoctadec-10-enoic acid; canrenone; isocorydine; santin; neochlorogenic acid; (±)-6-acetonyldihydrochelerythrine; and (10E,15E)-9,12,13-trihydroxyoctadeca-10,15-dienoic acid showed moderate to low log_2_fold values. Methanol extracts showed fewer key solvent- and plant part-associated metabolites including 4-decylbenzenesulfonic acid; 5,6,2′-trimethoxyflavone; canrenone; and neochlorogenic acid, while dichloromethane extracts showed even fewer metabolites such as 4-decylbenzenesulfonic acid, (-)-scoulerine, canrenone and quillaic acid. Only 5,6,2′-trimethoxyflavone was identified from water extracts. 4-decylbenzenesulfonic acid, isocorydine and santin (acetone extracts) showed the highest log_2_fold values of 9.53, 7.83 and 7.31, respectively.

**Figure 5:**
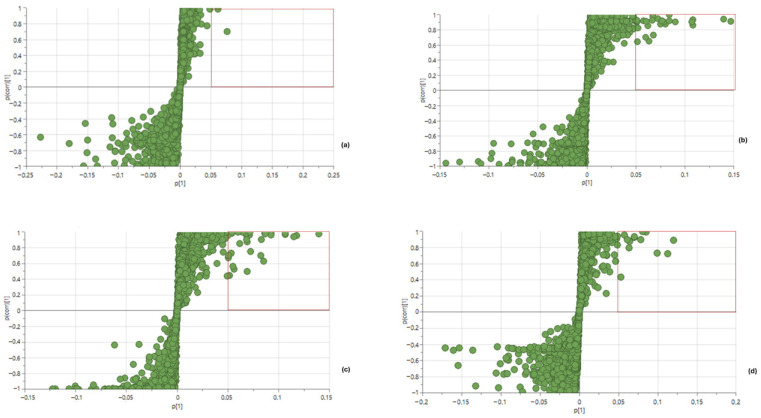
OPLS-DA-based S-plots showing differential distribution of key metabolic features (top right quadrant) in *Argemone mexicana* shoot and root water (**a**), methanol (**b**), acetone (**c**) and dichloromethane (**d**) extracts. Features located at the extremes of the plot (top right quadrant highlighted in red) represent key metabolic features with a strong contribution to the model.

Fourteen (14) metabolites were identified in *A. mexicana* extracts as extrapolated from S-plots Figure 5. Methanol extracts had the highest number of key metabolites with nine different metabolites such as feruloyltyramine, angoline and kaempferol-3-O-glucoside. Acetone extracts had eight metabolites including 4-decylbenzenesulfonic acid; feruloyltyramine; 5,6,2′-trimethoxyflavone; 9,12,13-trihydroxyoctadec-10-enoic acid; and isocorydine while dichloromethane extracts had five compounds, including 4-decylbenzenesulfonic acid, canrenone, and other metabolites. Water extracts had the lowest number of solvent and plant part-associated metabolites (2) such as gossypol and 9,12,13-trihydroxyoctadec-10-enoic acid (Table 2). Metabolites such as 9,12,13-trihydroxyoctadec-10-enoic acid, eleutheroside e1 and kaempferol-3-O-glucoside had the highest log_2_fold values of 5.16, 4.67 and 4.61, respectively.

**Figure 6:**
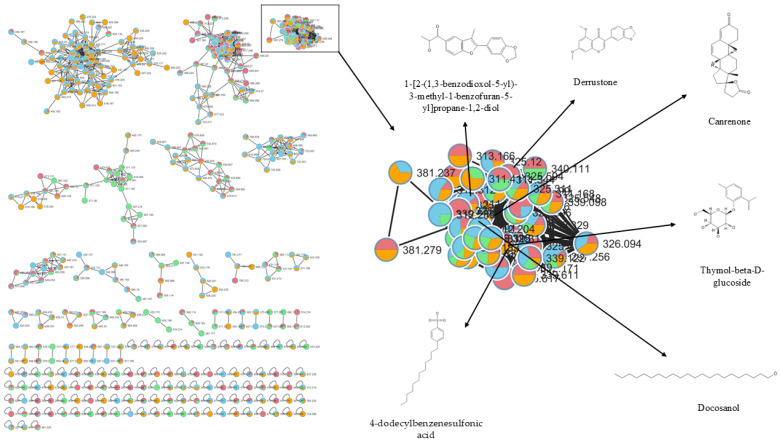
Molecular network of MS/MS spectra created in GNPS providing metabolome coverage from water extracts of *Argemone ochroleuca* shoot and root and *Argemone mexicana* shoot and root extract. *Argemone ochroleuca* shoot (red), *Argemone ochroleuca* root (blue), *Argemone mexicana* shoot (green) and *Argemone mexicana* root (orange) extracts including a zoomed-in solvent molecular network. Nodes from the chosen molecular class are identified with precursor mass and shown as pie charts depicting the ion intensities of *Argemone ochroleuca* and *Argemone mexicana* shoots and roots. Each depicted node represents a metabolite.

**Figure 7:**
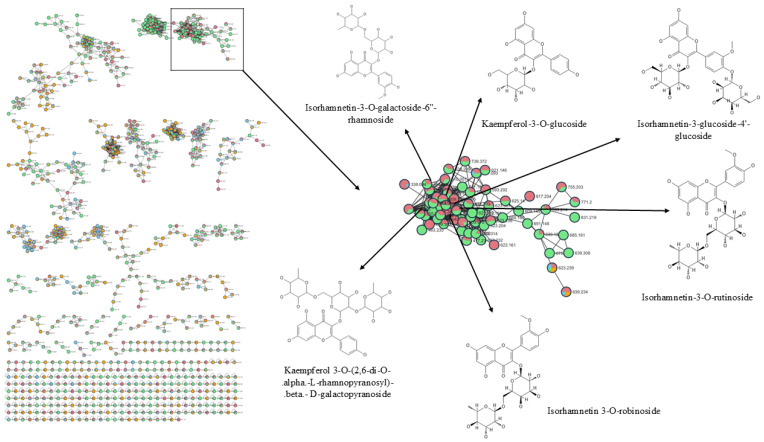
Molecular network of MS/MS spectra created in GNPS providing metabolome coverage from methanol extracts of *Argemone ochroleuca* shoot and root and *Argemone mexicana* shoot and root extract. *Argemone ochroleuca* shoot (red), *Argemone ochroleuca* root (blue), *Argemone mexicana* shoot (green) and *Argemone mexicana* root (orange) extracts including a zoomed-in solvent molecular network. Nodes from the chosen molecular class are identified with precursor mass and shown as pie charts depicting the ion intensities of *Argemone ochroleuca* and *Argemone mexicana* shoots and roots. Each depicted node represents a metabolite.

**Figure 8:**
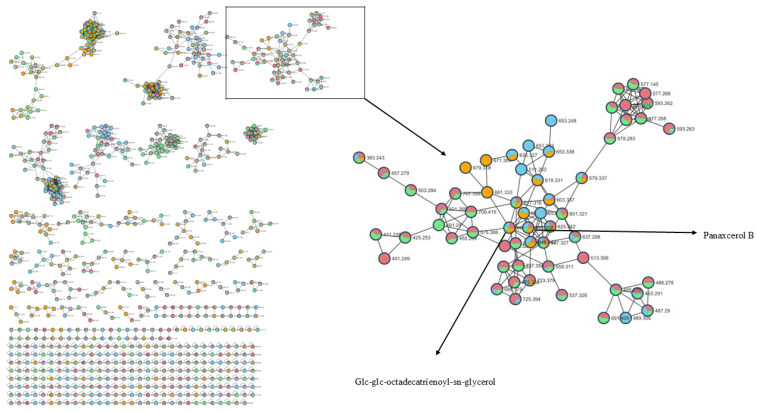
Molecular network of MS/MS spectra created in GNPS providing metabolome coverage from acetone extracts of *Argemone ochroleuca* shoot and root and *Argemone mexicana* shoot and root extract. *Argemone ochroleuca* shoot (red), *Argemone ochroleuca* root (blue), *Argemone mexicana* shoot (green) and *Argemone mexicana* root (orange) extracts including a zoomed-in solvent molecular network. Nodes from the chosen molecular class are identified with precursor mass and shown as pie charts depicting the ion intensities of *Argemone ochroleuca* and *Argemone mexicana* shoots and roots. Each depicted node represents a metabolite.

**Figure 9:**
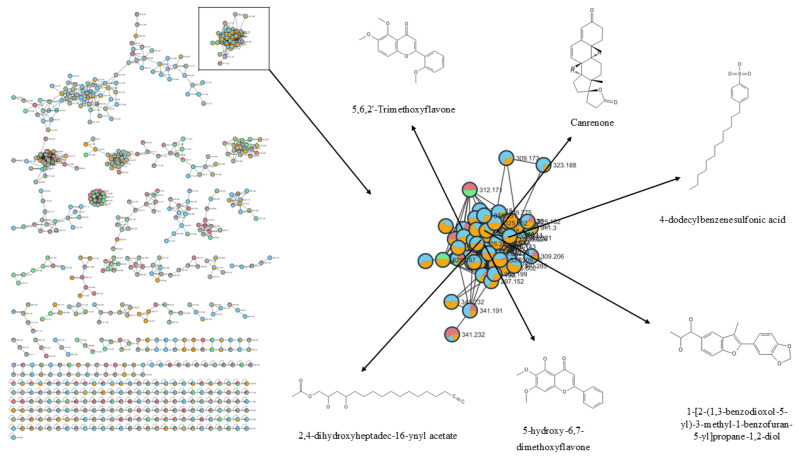
Molecular network of MS/MS spectra created in GNPS providing metabolome coverage from dichloromethane extracts of *Argemone ochroleuca* shoot and root and *Argemone mexicana* shoot and root extract. *Argemone ochroleuca* shoot (red), *Argemone ochroleuca* root (blue), *Argemone mexicana* shoot (green) and *Argemone mexicana* root (orange) extracts including a zoomed-in solvent molecular network. Nodes from the chosen molecular class are identified with precursor mass and shown as pie charts depicting the ion intensities of *Argemone ochroleuca* and *Argemone mexicana* shoots and roots. Each depicted node represents a metabolite.

Molecular networking analysis based on GNPS spectral matching revealed complex metabolite patterns in the extracts of *A. ochroleuca* and *A. mexicana*. Metabolites were computed into 461, 965, 885 and 655 nodes, where 352, 738, 627 and 480 metabolite nodes clustered into 36, 90, 78 and 64 molecular families for water (Figure 6), methanol (Figure 7), acetone (Figure 8) and dichloromethane (Figure 9) extracts, respectively. To be considered a molecular network metabolites need to be connected by two nodes at a minimum. However, 109, 227, 258 and 175 nodes were not grouped into a molecular family and were displayed as independent nodes at the base of the networks for water, methanol, acetone and dichloromethane extracts, respectively.

To evaluate the effect of different solvents on molecular networks and molecular families, certain molecular networks were studied (Figure 6, Figure 7, Figure 8 and Figure 9). In the flavonoid family, 5,6,2′-Trimethoxyflavone at *m*/*z* of 311.089 and derrustone at *m*/*z* 325.073 were detected in both plant species. This family also included structurally related metabolites belonging to different chemical classes, including fatty alcohol derivatives such as 2,4-dihydroxyheptadec-16-ynyl acetate (*m*/*z* 325.235), terpenoids derivatives such as canrenone (*m*/*z* 339.199), lignans such as Compound NP-012925 (*m*/*z* 325.1), and flavonoids such as 5-hydroxy-6,7-dimethoxyflavone (*m*/*z* 297.075). Additionally, a macrolide-like metabolite compound NP-008285 at *m*/*z* 325.142 clustered into the same molecular family, suggesting structural relatedness among these metabolites.

Isorhamnetin flavonoids and structurally similar metabolites were clustered into the same molecular family (Figure 7). These included isorhamnetin-3-glucoside-4′-glucoside (*m*/*z* 639.156), isorhamnetin-3-O-rutinoside (*m*/*z* 623.204), isorhamnetin-3-O-galactoside-6″-rhamnoside (*m*/*z* 623.161), kaempferol-3-O-glucoside (*m*/*z* 447.092), isorhamnetin 3-O-robinoside (*m*/*z* 669.166) and kaempferol 3-O-(2,6-di-O-.alpha.-L-rhamnopyranosyl)-.beta.-D-galactopyranoside (*m*/*z* 739.209) together with several related spectral nodes.

Lipid-derived metabolites also formed distinct molecular families. For example, Glc-glc-octadecatrienoyl-sn-glycerol (*m*/*z* 721.364), detected in methanol, acetone and dichloromethane extracts, clustered with the structurally related metabolite panaxcerol B (*m*/*z* 559.311) (Figure 8). Several additional nodes within this molecular family (*m*/*z* 383.243, 425.253, 537.326, 579.337, 725.394) could not be annotated using GNPS spectral library, suggesting the presence of potentially uncharacterized metabolites. Overall, molecular networking revealed chemically diverse metabolite profiles in both *Argemone* species, with flavonoids, lipid derivatives, terpenoids and lignans forming prominent molecular families across the different solvent extracts.

To further characterize the metabolites represented within the molecular networks, annotation tools and spectral libraries within GNPS were used for putative metabolite identification. Based on the spectral library matching, a total of 59 and 54 metabolites were putatively annotated from the extracts of *A. ochroleuca* extracts (Table 3) and *A. mexicana* (Table 4), respectively. The highest numbers of metabolites were identified from methanol extracts with 26 and 18 metabolites annotated from the shoots and roots of *A. ochroleuca* and 22 and 17 metabolites from *A. mexicana* shoots and roots, followed by acetone extracts with 24 and 16 metabolites identified from *A. ochroleuca* shoots and roots and 20 and 15 metabolites from *A. mexicana* shoots and roots. Water extracts yielded 14 and 12 metabolites from *A. ochroleuca* shoots and roots and 13 and 11 metabolites from *A. mexicana* shoots and roots and dichloromethane extracts had the lowest number of metabolites identified from both plants with 12 and 10 metabolites annotated from *A. ochroleuca* and 14 and 12 from *A. mexicana* shoots and roots, respectively. As evidenced by the number of annotated metabolites, shoot extracts had a higher diversity of metabolites identified from both species in comparison to root extracts across all solvents.

The similarities in metabolite composition are from the presence of common metabolites in both plants; however, there are a few metabolites that were unique to each plant species. It was also observed that 4-decylbenzenesulfonic acid (*m*/*z* 297.152) and 4-dodecylbenzenesulfonic acid (*m*/*z* 325.184) were present in all water, acetone, methanol and dichloromethane of *A. ochroleuca* and *A. mexicana* extracts, respectively. The results also revealed that the plant species are rich in flavonoids, among which were rutin (*m*/*z* 609.145) and luteolin (*m*/*z* 285.04) observed in both *A. ochroleuca* and *A. mexicana* extracts.

Although a similar metabolomic profile was observed between *A. ochroleuca* and *A. mexicana* water, methanol, acetone and dichloromethane plant extracts, there were metabolites that were identified from either plant species such as abscisic acid *m*/*z* 263.128 identified from *A. ochroleuca* methanol and acetone shoot extracts and eriodictyol *m*/*z* 287.055 and isorhamnetin *m*/*z* 315.05 identified from *A. mexicana* methanol shoot extracts. Other metabolites were santin at *m*/*z* 343.081 identified from *A. ochroleuca* acetone shoot extract and tsangane L 3-glucoside at *m*/*z* 395.204 *A. ochroleuca* water (root) and acetone (shoot) extracts. The differences in metabolite distribution across the two plant species, different solvents and plant parts could be attributed to different factors like the environment where they were growing and the phytochemical differences between the two plant species. Other metabolites were identified from both plant species, however differed across the different solvents and plant parts. For example, 4-Decylbenzenesulfonic acid identified from all *A. ochroleuca* extracts across all different solvents and plant parts; however, in *A. mexicana*, the same metabolite was identified in the other extracts except for water shoot and root and dichloromethane shoot extract. This distribution points to the fact that metabolites are plant part- and solvent-specific.

**Figure 10:**
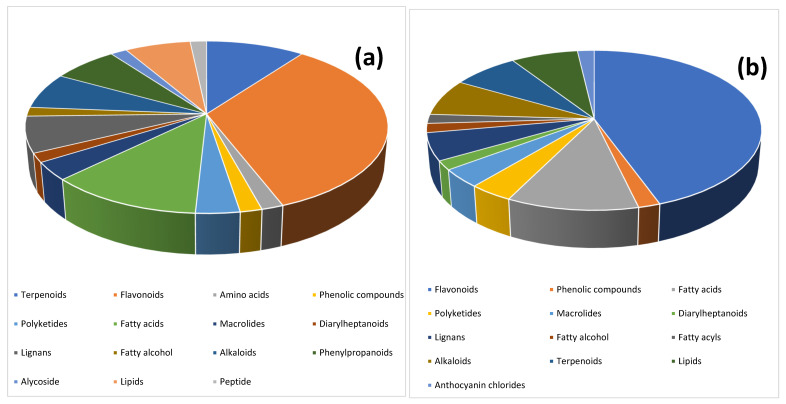
*Argemone ochroleuca* (**a**) and *Argemone mexicana* (**b**) compound classes of the identified metabolites.

Different metabolite classes were identified from *A. ochroleuca* and *A. mexicana* water, methanol, acetone and dichloromethane extracts. It was observed that these plant species contain 15 and 13 different metabolite classes for both *A. ochroleuca* and *A*. *mexicana*. Flavonoids were the most abundant class in all extracts, followed by fatty acids, terpenoids, alkaloids, and phenylpropanoids, while other groups such as lignans, lipids, macrolides, and peptides were less abundant. Different chemical classes such as terpenoids (canrenone), flavonoids (5,6,2′-Trimethoxyflavone), amino acids (n-fructosyl pyroglutamate), phenolic compounds (6-gingerol), polyketides (4-Decylbenzenesulfonic acid), fatty acids (9,12,13-trihydroxyoctadec-10-enoic acid), macrolides (pyrenophorol), diarylheptanoids (feruloyltyramine), lignans (1-[2-(1,3-benzodioxol-5-yl)-3-methyl-1-benzofuran-5-yl]propane-1,2-diol), fatty alcohols (2,4-dihydroxyheptadec-16-ynyl acetate), alkaloids [(-)-scoulerine], phenylpropanoids (neochlorogenic acid), alycosides (tsangane L 3-glucoside), lipids (1-oleoyl-L-.alpha.-lysophosphatidic acid) and peptides (eleutherazine B) were identified from *A. ochroleuca* extracts (Figure 10a). Similarly, flavonoids (kaempferol-3-O-glucoside), phenolic compounds (6-gingerol), fatty acids (9-hydroxy-10,12-octadecadienoic acid), polyketides (4-dodecylbenzenesulfonic acid), terpenoids (gossypol), macrolides (compound NP-008285), diarylheptanoids (feruloyltyramine), lignans (1-[2-(1,3-benzodioxol-5-yl)-3-methyl-1-benzofuran-5-yl]propane-1,2-diol), fatty alcohols (2,4-dihydroxyheptadec-16-ynyl acetate), fatty acyls (docosanol), alkaloids (isocorydine), glycerophospholipids, anthocyanin chlorides (cyanidin-3,5-di-O-glucoside chloride) and lipids [1-Hexadecanoyl-sn-glycero-3-phospho-(1′-myo-inositol)] were identified from *A. mexicana* (Figure 10b).

## 3. Discussion

Untargeted LC-MS-based metabolomics coupled with molecular networking enabled the characterization of the metabolite profiles of root and shoot extracts of *A. ochroleuca* and *A. mexicana*. This approach proved effective for metabolite profiling, as multivariate data analysis can pinpoint metabolites responsible for observed bioactivity without first requiring prior compound isolation [8,23,24,25]. The integration of LC-MS/MS data with molecular networking in this study further facilitated metabolite annotation by grouping compounds with similar fragmentation patterns, thereby improving the identification of structurally related metabolites [21]. The application of this integrated approach revealed substantial variation in metabolite composition between the two *Argemone* species, as well as differences associated with plant part and extraction solvent.

Species of the genus *Argemone* are well known for their rich secondary metabolites. In agreement with this, *Argemone ochroleuca* displayed a more diverse metabolite profile than *A. mexicana*, as shown by the larger number of metabolites identified across numerous classes. Such differences are not unexpected, as metabolomic studies have shown that metabolic profiles are often taxon-dependent, reflecting species-specific biosynthetic pathways and regulatory mechanisms [26]. Plants within the same family may produce similar classes of metabolites due to shared biosynthetic routes and regulatory enzymes. However, differences in the abundance and diversity of these compounds can occur among species [27]. Metabolite composition also varied according to extraction solvent and plant part. Methanol and acetone extracts had a higher number of metabolites compared to water and dichloromethane extracts, suggesting that polar solvents are more efficient in extracting diverse metabolites from *A. ochroleuca* and *A. mexicana*. Furthermore, most metabolites were detected in shoot extracts. Variations in metabolite distribution among plant parts have been widely reported and are often attributed to tissue-specific biosynthesis, developmental regulation, and the specialized ecological functions of secondary metabolites [28,29]. These compounds frequently accumulate in particular tissues where they contribute to defence, stress responses, or interactions with pollinators and other organisms [30]. Different classes of secondary metabolites including flavonoids, fatty acids, terpenoids, alkaloids and others from different plant parts were observed in this study.

Flavonoids were identified as the main classes for both *A. ochroleuca* and *A. mexicana* extracts in this study. Khan and Bhadauria [10] reported a wide range of flavonoid content, phenolic content, and fatty acids in *A. mexicana* extracts. In exploring the metabolomic profile of *A. mexicana* leaf extracts using LC-MS, various metabolites belonging to alkaloid and flavonoid classes were identified from aqueous, acetone, hexane and ethanolic extracts [8]. These metabolites have been associated with diverse biological activities. Flavonoids such as rutin [31,32,33], quercetin [34] and luteolin have been reported to have antibacterial activity [24]. Kaempferol, kaempferol-3-*O*-rutinoside, luteolin, apigenin, quercetin, rutin, isorhamnetin and other flavonoids were reported in the roots and shoots of these two plant species and have shown significant antibacterial activity on phytopathogenic bacteria [35].

Alkaloids were also identified from both *A. ochroleuca* and *A. mexicana* extracts. Reyes-Luna et al. [4] profiled *Argemone ochroleuca* extracts using gas chromatography–mass spectrometry (GC-MS) and observed that the extracts were rich in alkaloids, terpenes and phenolic compounds. Alkaloids such as (-)-scoulerine, isocorydine and (±)-6-acetonyldihydrochelerythrine were reported from *A. mexicana* extracts [36,37], with some having antifungal activity on phytopathogens [38,39,40]. Other chemical classes such as terpenoids, amino acids, polyketides, fatty acids, lignans, fatty alcohols, and lipids were amongst others identified from the extracts. Terpenoids such as quillaic acid, canrenone and gossypol were identified as key metabolites in this study, whereas previous studies have attributed these phytochemicals to the antibacterial activity observed on Gram-positive and Gram-negative bacteria [41,42,43,44].

Phytochemicals identified in the current study have previously been reported to demonstrate antifungal activity against phytopathogens such as *Fusarium oxysporum* and *Sclerotinia sclerotiorum* [6,9,45], as well as antibacterial, anti-inflammatory, antifungal and antimycotic activities, highlighting the potential pharmacological relevance of these plant extracts [10,45,46]. In addition to the annotated metabolites, several spectral features could not be confidently matched to existing databases, indicating that *A. ochroleuca* and *A. mexicana* may contain additional, yet-to-be-characterized compounds. Consistent with the principles of molecular networking, some clusters may contain structurally related but unannotated metabolites, while other nodes occurred as singletons, suggesting further chemical complexity within these species, as suggested by Ndou et al. [21]. Future improvements in spectral databases and the availability of reference standards may facilitate the annotation of these currently unidentified metabolites.

Collectively, the metabolic profiles of *A. ochroleuca* and *A. mexicana* demonstrate remarkable biochemical diversity and provide a foundation for future functional and comparative studies. The abundance of flavonoids, alkaloids, terpenoids and other bio-active metabolites further highlights the potential of these species as valuable sources of natural products for agricultural, medicinal and biotechnological applications. Although no bioassays were reported in this study, LC-MS profiling provides a foundation for future investigations into possible applications of these metabolites.

## 4. Materials and Methods

### 4.1. Collection of Plant Material

*Argemone ochroleuca* Sweet and *Argemone mexicana* Linn were collected from Mpumalanga Province in South Africa. *Argemone ochroleuca* was collected from the Agricultural Research Council (ARC), Mbombela (25.4518° S, 30.9697° E), whereas *A. mexicana* was collected at Bushbuckridge, Mkhuhlu (24.96646° S, 31.26544° E). These plants were authenticated at the H.G.W.J. Schweickerdt Herbarium (PRU), University of Pretoria, and assigned accession numbers 132731 for *A. ochroleuca* and 132732 for *A. mexicana*.

### 4.2. Preparation of Plant Extracts

Dried shoots and roots of *A. ochroleuca* and *A. mexicana* were extracted with solvents of different polarity: distilled water, acetone, methanol or dichloromethane. The extracts were prepared by extracting 20 g of *A. ochroleuca* and *A. mexicana* shoot and root plant material separately with 200 mL of distilled water, acetone, methanol or dichloromethane. The resultant mixtures were then shaken in an orbital shaker at 130 rpm for 24 h. A Buchner funnel lined with Whatman filter No. 1 paper was used to filter the extracts [47]. The collected organic extracts were concentrated using a rotary evaporator (Bϋchi, Flawil, Switzerland) and further air-dried in a fume hood to obtain the crude extracts. Aqueous extracts were freeze-dried using a freeze drier (Alpha 1-2 Ldplus (Lasec SA (Pty) Ltd., Cape Town, South Africa) until they were completely dried, resulting in 16 different extracts. The extracts were stored in a refrigerator at −4 °C until they were used.

### 4.3. Metabolite Extraction

Metabolite extraction was carried out on the prepared 16 plant extracts from the roots and shoots of *A. ochroleuca* and *A. mexicana* extracted using water, acetone, methanol and dichloromethane. Samples were prepared following a modified method by Ramabulana et al. [48]. Briefly, 50 mg of the crude extracts was weighed into 1.5 mL Eppendorf tubes and mixed with 1.5 mL of 80% cold methanol (LC-MS grade) mixed with 20% sterile distilled water. The samples were dissolved using a sonicator for 1 h at 25 (±2) °C. The samples were then centrifuged for 5 min at 4000 rpm. The supernatants were filtered using nylon filters (0.22 μm) (Thermo Fisher, Johannesburg, South Africa) to remove debris and the filtered extracts were put into glass vials for chromatography analysis that had 500 μL inserts and sealed. The samples were kept at 4 °C until they could be analyzed further.

### 4.4. LC-QTOF-MS Analysis

Metabolite separation and identification were performed using a liquid chromatography–quadrupole time-of-flight tandem mass spectrometry system (LCMS-9030 qTOF, Shimadzu Corporation, Kyoto, Japan) at the University of Venda, Centre of Excellence in Mass Spectrometry for Southern Africa (CEMSSA), following the method detailed by Ramabulana et al. [48]. The chromatographic separation was achieved using a Evosphere C18 column (100 × 2.1 mm, 1.7 µm particle size), maintained at 55 °C. The samples were injected twice at a volume of 3 µL using a binary solvent system made up of solvent A (Milli-Q water with 0.1% formic acid, HPLC grade, Merck, Darmstadt, Germany) and solvent B (methanol with 0.1% formic acid, UHPLC grade, Romil SpS, Cambridge, UK), pumped at a flow rate of 0.4 mL/min. The 13 min gradient technique began at 5% B, was held isocratically for 3 min, and then returned to 5% B over 2 min before reaching 95% B in 7 min, maintaining it for 4 min, and then returning to 5% B in 2 min, followed by a 2 min re-equilibration period. The detection was carried out utilizing qTOF mass spectrometry in negative electrospray ionization mode with data-dependent acquisition (DDA). The key operating settings were a 4.0 kV interface voltage, 300 °C interface temperature, 3 L/min gas flow, 400 °C heat block, 280 °C DL temperature, 1.8 kV detector voltage, and 42 °C flight tube temperature. Ion fragmentation was carried out using argon gas using collision-induced dissociation (CID) at 30 eV with a 5 eV spread.

### 4.5. Data Pre-Processing and Multivariate Data Analysis

The obtained raw LC-MS data included retention times, mass spectra and peak intensities. The MzML files were pre-processed using XCMS online (https://xcmsonline.scripps.edu, accessed 3 March 2025), with UPLC/UHD Q-TOF negative mode parameters using the centWave technique for feature detection according to a method detailed in Ramabulana et al. [48] and Makhubu et al. [49]. The maximal tolerated *m*/*z* deviation in consecutive scans was set at 15 ppm, the signal-to-noise ratio was set at 6, the prefilters peak was set at 3 and the intensity was set at 700. Retention time correction was performed using the obiwarp technique with a profStep of 1. Peak alignment was performed using a minimum fraction of 0.5 across all samples and an mzwid grouping width of 0.025. Following a post hoc analysis and a Kruskal–Wallis non-parametric statistical test, the data were normalized using the median fold change.

The feature files obtained from XCMS online included a total of 11,651 features from the comparison between *A. ochroleuca* and *A. mexicana*. For *A. ochroleuca* extracts, the following feature list was detected: 2992 (water AOS vs. AOR), 4644 (methanol AOS vs. AOR), 5073 (acetone AOS vs. AOR) and 3613 (dichloromethane AOS vs. AOR) features for *A. ochroleuca* extracts; similarly, 1871 (water AMS vs. AMR), 5637 (methanol AMS vs. AMR), 4467 (acetone AMS vs. AMR) and 2490 (dichloromethane AMS vs. AMR) features were detected for *A. mexicana* extracts. MetaboAnalyst 6.0 (https://www.metaboanalyst.ca/ (accessed on 9 March 2025)) was used for chemometric analysis. Data processing included sample normalization, log transformation, median normalization, data integrity checks, missing value estimates, and Pareto scaling of databases. Following data processing and standardization, principal component analysis (PCA) was used to evaluate the data. Soft Independent Modeling of Class Analogy (Simca^®^ 18) software (Sartorius, Johannesburg, South Africa) was used to generate an Orthogonal Partial Least Squares—Discriminant Analysis (OPLS-DA) loading S-plot showing the differential distribution of metabolites. These were annotated by comparing their retention times and spectral features with databases, which led to their probable identification.

### 4.6. Metabolite Annotation

The acquired raw datasets obtained from the Shimadzu LCMS-9030-qTOF-MS were converted to open-source format (mzML) files which were then imported to the Global Natural Products Social Molecular Networking (GNPS) online platform for Feature-Based Molecular Networking (FBMN) via WinSCP 6.5.3 software to determine the distinctive molecular networks of metabolites based on their MS/MS spectra and metabolite annotation [50]. The computation parameters for FBMN were set as: precursor ion mass tolerance and fragment ion mass tolerance of 0.02 Da for both aspects. To analyze spectral similarity, a minimum pairs cosine of 0.6, minimum matched fragment ions of 4 and network TopK of 50 was set. A library minimum search match of 4 was also set. To visualize and analyze the computed networks, the Cytoscape network visualization software (version 3.10.3) was used. Manual annotations were performed using the existing published literature on the phytochemical analysis of the plants and retention times. Annotations were validated and confirmed using PubChem (https://pubchem.ncbi.nlm.nih.gov/) and against the existing literature.

## 5. Conclusions

The present study offers insight into the metabolomic profiles of *A. ochroleuca* and *A. mexicana* across water, methanol, acetone, and dichloromethane extracts. Overall, *A. ochroleuca* had the highest metabolite abundance, particularly in methanol and acetone extracts and in shoot extracts. Metabolomic profiling revealed a diverse range of metabolites in both species, including flavonoids, alkaloids, terpenoids, phenolic compounds, fatty acids, polyketides and monoterpenoids, with flavonoids representing the dominant class. Representative compounds identified in this study include alkaloids such as scoulerine and isocorydine, terpenoids such as quillaic acid, canrenone, and gossypol, and flavonoids such as quercetin, rutin, luteolin, kaempferol-3-O-glucoside, and apigenin. Several of these compounds belong to widely distributed plant metabolite classes and have been previously associated with antibacterial and antifungal activities, supporting the biological relevance of the metabolite profiles observed in this study.

These findings demonstrate that the integration of LC-MS-based metabolomics and molecular networking, together with solvent- and plant part-specific extraction methods, enhances the characterization of chemically diverse metabolites in *A*. *ochroleuca* and *A. mexicana*. Due to the use of negative ionization mode only, the present analysis may not fully capture the complete metabolite profile of these species. Future studies incorporating positive ionization mode and complementary techniques such as GC-MS may therefore provide broader metabolite coverage. Furthermore, the majority of the detected metabolites have not previously been reported from these two species, highlighting their chemical novelty and supporting their value as underexplored sources of bioactive compounds. This study therefore provides a foundation for future work aimed at investigating the individual and combined biological activities of these metabolites for potential agricultural and pharmaceutical applications.

## Figures and Tables

**Table 1:** *Argemone ochroleuca* key metabolites identified from S-plot loadings of shoot and root extracts obtained using water, methanol, acetone, and dichloromethane along with their log_2_fold changes.

Metabolite	Class	Precursor *m*/*z*	Molecular Formula	RT (min)	Fragment Ions	Water	Methanol	Acetone	Dichloromethane
6-Gingerol	Phenolic compounds	293.175	C_17_H_26_O_4_	8.66	99.08 293.18	ND	ND	**4.05**	ND
4-decylbenzenesulfonic acid	Polyketides	297.152	C_16_H_26_O_3_S	7.98	297.15 298.15	ND	1.07	**9.5** **3**	1.29
5,6,2′-Trimethoxyflavone	Flavonoids	311.089	C_18_H_16_O_5_	5.24	183.01 311.17	0.89	0.81	3.73	1.36
Feruloyltyramine	Diarylheptanoids	312.123	C_18_H_19_NO_4_	5.48	147.04 148.05 178.05	ND	ND	**5.90**	1.87
1-[2-(1,3-benzodioxol-5-yl)-3-methyl-1-benzofuran-5-yl]propane-1,2-diol	Lignans	325.096	C_19_H_18_O_5_	8.74	183.01 197.03	ND	0.65	2.36	1.40
(-)-scoulerine	Alkaloids	327.574	C_19_H_21_NO_4_	6.48	327.22 328.22	ND	ND	ND	2.00
9,12,13-trihydroxyoctadec-10-enoic acid	Fatty acids	329.232	C_18_H_34_O_5_	6.71	171.10 211.13 329.23	ND	1.01	**4.77**	ND
Canrenone	Terpenoids	339.199	C_22_H_28_O_3_	9.10	183.01 33.02	ND	0.83	2.44	1.70
Isocorydine	Alkaloids	341.232	C_20_H_23_NO_4_	5.66	341.20 342.20	ND	ND	**7.83**	ND
Santin	Flavonoids	343.081	C_18_H_16_O_7_	7.27	270.02	ND	ND	**7.31**	ND
Neochlorogenic acid	Phenylpropanoids	353.087	C_16_H_18_O_9_	1.95	191.06	ND	1.60	**5.13**	ND
(±)-6-Acetonyldihydrochelerythrine	Alkaloids	405.248	C_24_H_23_NO_5_	5.44	405.25	ND	ND	**6.25**	ND
Quillaic acid	Terpenoids	485.326	C_30_H_46_O_5_	7.72	485.33	ND	ND	ND	2.11
(10E,15E)-9,12,13-trihydroxyoctadeca-10,15-dienoic acid	Fatty acids	677.423	C_18_H_32_O_5_	6.53	171.10 183.14 233.12 327.22	ND	ND	2.14	3.95

ND: not detected. RT: retention time. The criteria used for log_2_fold values were: very high, >4; high, 3–4; moderate, 1–2. Bolded log_2_fold values represent the highest values.

**Table 2:** *Argemone mexicana* key metabolites identified from S-plot loadings of shoot and root extracts obtained using water, methanol, acetone, and dichloromethane along with their log_2_fold changes.

Metabolite	Class	Precursor *m*/*z*	Molecular Formula	RT (min)	Fragment Ions	Water	Methanol	Acetone	Dichloromethane
9-hydroxy-10,12-octadecadienoic acid	Fatty acids	295.227	C_18_H_32_O_3_	8.25	171.10 277.22 295.23 296.23	ND	2.57	ND	ND
4-decylbenzenesulfonic acid	Polyketides	297.152	C_16_H_26_O_3_S	7.98	297.15 298.15	ND	ND	**4.47**	1.37
5,6,2′-Trimethoxyflavone	Flavonoids	311.089	C_18_H_16_O_5_	5.24	183.01 311.17	ND	0.84	0.31	1.22
Feruloyltyramine	Diarylheptanoids	312.123	C_18_H_19_NO_4_	5.48	147.04 148.05 178.05	ND	1.92	2.54	1.73
1-[2-(1,3-benzodioxol-5-yl)-3-methyl-1-benzofuran-5-yl]propane-1,2-diol	Lignans	325.096	C_19_H_18_O_5_	8.74	183.01 197.03	ND	ND	0.38	1.21
4-dodecylbenzenesulfonic acid	Polyketides	325.184	C_18_H_30_O_3_S	8.75	325.18	ND	0.85	ND	ND
9,12,13-trihydroxyoctadec-10-enoic acid	Fatty acids	329.232	C_18_H_34_O_5_	6.71	171.10 211.13 329.23	**5.16**	1.32	1.51	ND
Canrenone	Terpenoids	339.199	C_22_H_28_O_3_	9.10	183.01 33.02	ND	0.97	0.60	1.79
Isocorydine	Alkaloids	341.232	C_20_H_23_NO_4_	5.66	341.20 342.20	ND	ND	1.28	ND
Angoline	Alkaloids	379.171	C_22_H_21_NO_5_	1.32	379.08	ND	2.05	ND	ND
Kaempferol-3-O-glucoside	Flavonoids	447.092	C_21_H_20_O_11_	5.49	227.03 255.03 284.03	ND	**4.61**	ND	ND
Gossypol	Terpenoids	517.185	C_30_H_30_O_8_	9.05	259.10 471.18 489.19 499.18 517.19	3.83	ND	ND	ND
Eleutheroside e1	Lignans	579.208	C_28_H_36_O_13_	5.45	181.05 402.12 417.15	ND	**4.67**	ND	ND
(10E,15E)-9,12,13-trihydroxyoctadeca-10,15-dienoic acid	Fatty acids	677.423	C_18_H_32_O_5_	6.53	171.10 183.14 233.12 327.22	ND	ND	1.17	ND

ND: not detected. RT: retention time. The criteria used for log_2_fold values were: very high, >4; high, 3–4; moderate, 1–2. Bolded log_2_fold values represent the highest values.

**Table 3:** Putatively annotated metabolites detected in *Argemone ochroleuca* extracts.

S/NO.	Metabolite	Class	Precursor *m*/*z*	Adduct	Molecular Formula	RT (min)	Fragment Ions	Water		Methanol		Acetone		Dichloromethane	
								Shoot	Root	Shoot	Root	Shoot	Root	Shoot	Root
1	Abscisic acid	Terpenoids	263.128	[M − H]^−^	C_15_H_20_O_4_	5.76	122.04 136.05 203.11	−	−	+	−	+	−	−	−
2	Apigenin	Flavonoids	269.045	[M − H]^−^	C_15_H_10_O_5_	4.04	117.03 269.04	−	−	+	−	−	−	−	−
3	Luteolin	Flavonoids	285.04	[M − H]^−^	C_15_H_10_O_6_	5.93	133.03 151.00 285.04	−	−	+	−	−	−	−	−
4	2-(3,4-Dihydroxyphenyl)-5,7-Dihydroxy-4h-Chromen-4-One	Flavonoids	285.04	[M − H]^−^	C_15_H_10_O_6_	5.97	133.03	−	−	−	−	+	−	−	−
5	N-Fructosyl pyroglutamate	Amino acids	290.088	[M − H]^−^	C_11_H_17_NO_8_	0.93	128.04 200.06 201.06 212.06 290.09	−	−	+	−	−	−	−	−
6	6-Gingerol	Phenolic compounds	293.175	[M − H]^−^	C_17_H_26_O_4_	8.66	99.08 293.18	−	−	+	−	−	−	−	−
7	5-hydroxy-6,7-dimethoxyflavone	Flavonoids	297.087	[M − H]^−^	C_17_H_14_O_5_	8.06	183.01 239.03	−	−	−	+	+	−	−	+
8	4-Decylbenzenesulfonic acid	Polyketides	297.152	[M − H]^−^	C_16_H_26_O_3_S	7.98	297.15 298.15	+	+	+	+	+	+	+	+
9	5,6,2′-Trimethoxyflavone	Flavonoids	311.089	[M − H]^−^	C_18_H_16_O_5_	5.24	183.01 311.17	+	+	−	+	+	+	−	+
10	Thymol-beta-D-glucoside	Terpenoids	311.168	[M − H]^−^	C_16_H_24_O_6_	8.40	149.10 183.01 197.03 311.17	+	+	+	+	−	+	−	−
11	Pyrenophorol	Macrolides	311.169	[M − H]^−^	C_16_H_24_O_6_	8.06	183.01 197.03	−	−	−	−	+	−	+	+
12	Feruloyltyramine	Diarylheptanoids	312.123	[M − H]^−^	C_18_H_19_NO_4_	5.48	147.04 148.05 178.05	−	−	+	+	+	+	+	+
13	Derrustone	Flavonoids	325.073	[M − H]^−^	C_18_H_14_O_6_	3.90	183.01 311.17 325.18	+	+	−	−	−	+	−	−
14	1-[2-(1,3-benzodioxol-5-yl)-3-methyl-1-benzofuran-5-yl]propane-1,2-diol	Lignans	325.096	[M − H]^−^	C_19_H_18_O_5_	8.74	183.01 197.03	+	+	+	+	+	+	+	+
15	Compound NP-008285	Macrolides	325.142	[M − H]^−^	C_16_H_22_O_7_	8.75	183.01	+	+	+	−	−	−	−	−
16	4-dodecylbenzenesulfonic acid	Polyketides	325.184	[M − H]^−^	C_18_H_30_O_3_S	8.75	325.18	+	+	+	+	+	+	+	−
17	9-E1t-PhytoP	Fatty acids	325.20	[M − H]^−^	C_18_H_30_O_5_	6.20	289.18 307.19 325.20	−	−	−	−	+	−	−	−
18	2,4-dihydroxyheptadec-16-ynyl acetate	Fatty alcohol	325.258	[M − H]^−^	C_19_H_34_O_4_	8.75	79.96 183.01	−	−	−	−	+	+	−	+
19	9-epi-9-F1t-PhytoP	Fatty acids	327.217	[M − H]^−^	C_18_H_32_O_5_	6.55	171.10 327.22	−	−	−	−	+	−	−	+
20	9,12,13-trihydroxyoctadeca-10,15-dienoic acid	Fatty acids	327.217	[M − H]^−^	C_18_H_32_O_5_	6.54	211.13 229.15 327.22	−	−	−	+	−	−	−	+
21	16-epi-16-F1t-PhytoP	Fatty acids	327.22	[M − H]^−^	C_18_H_32_O_5_	5.98	283.19 327.22	−	−	−	−	−	+	−	−
22	(-)-scoulerine	Alkaloids	327.574	-	C_19_H_21_NO_4_	6.48	327.22 328.22	+	+	+	+	+	−	+	−
23	9,12,13-trihydroxyoctadec-10-enoic acid	Fatty acids	329.232	[M − H]^−^	C_18_H_34_O_5_	6.71	171.10 211.13 329.23	−	−	+	+	+	+	−	−
24	[5-acetyloxy-3-(hydroxymethyl)-2-oxo-6-propan-2-ylcyclohex-3-en-1-yl] 3-methylpentanoate	Terpenoids	339.165	[M − H]^−^	C_18_H_28_O_6_	7.01	183.01	−	−	−	−	+	−	−	−
25	Canrenone	Terpenoids	339.199	[M − H]^−^	C_22_H_28_O_3_	9.10	183.01 33.02	+	+	+	+	+	+	+	+
26	Isocorydine	Alkaloids	341.232	-	C_20_H_23_NO_4_	5.66	341.20 342.20	+	−	−	−	+	+	+	+
27	Santin	Flavonoids	343.081	[M − H]^−^	C_18_H_16_O_7_	7.27	270.02	−	−	−	−	+	−	−	−
28	Neochlorogenic acid	Phenylpropanoids	353.087	[M − H]^−^	C_16_H_18_O_9_	1.95	191.06	+	+	−	+	−	+	−	−
29	4-Caffeoylquinic acid	Phenylpropanoids	353.12	[M − H]^−^	C_16_H_18_O_9_	5.40	135.05 173.03 179.04 191.06 218.97 353.10	−	−	+	−	−	+	−	−
30	Tsangane L 3-glucoside	Alycoside	395.204	M+Na-2H	C_19_H_34_O_7_	6.54	171.10 193.08 233.11 327.22	−	+	+	−	−	−	−	−
31	Angoline	Alkaloids	379.171	-	C_22_H_21_NO_5_	1.32	379.08	−	+	+	−	−	−	+	−
32	1-O-β-D-glucopyranosyl sinapate	Phenylpropanoids	385.114	[M − H]^−^	C_17_H_22_O_10_	4.62	175.00 193.03 205.05 223.06 385.11	−	−	−	−	+	−	−	−
33	(±)-6-Acetonyldihydrochelerythrine	Alkaloids	405.248	-	C_24_H_23_NO_5_	5.44	405.25	−	−	−	−	+	+	+	+
34	1-Oleoyl-L-.alpha.-lysophosphatidic acid	Lipids	435.249	[M − H]^−^	C_21_H_41_O_7_P	9.33	153.00 435.26	−	−	−	−	−	−	−	+
35	Kaempferol-3-O-glucoside	Flavonoids	447.092	[M − H]^−^	C_21_H_20_O_11_	5.49	227.03 255.03 284.03	−	−	+	−	+	−	−	−
36	Lysophosphatidylglycerol	Lipids	483.272	[M − H]^−^	C_22_H_44_O_9_P^−^	9.23	255.22 483.37	−	−	+	−	−	−	−	−
37	Eleutherazine B	Peptides	451.256	[M − H]^−^	C_22_H_36_N_4_O_6_	4.52	108.04 155.12 165.10 227.15 309.19 339.20 391.23 421.25	+	−	+	−	+	−	−	−
38	Isorhamnetin 3-galactoside	Flavonoids	477.103	[M − H]^−^	C_22_H_22_O_12_	5.62	243.03 271.02 285.04 314.04 477.10	−	−	+	−	+	−	−	−
39	Quillaic acid	Terpenoids	485.326	[M − H]^−^	C_30_H_46_O_5_	7.72	485.33	−	−	−	−	−	−	−	+
40	Gossypol	Terpenoids	517.185	[M − H]^−^	C_30_H_30_O_8_	9.05	259.10 471.18 489.19 499.18 517.19	+	+	−	−	−	−	−	−
41	D-glucopyranosyl}oxy)-3-methoxyphenyl]acrylic acid	Phenylpropanoids	533.13	M+FA-H	C_21_H_28_O_13_	3.79	193.05 487.15	+	−	−	−	−	−	−	−
42	Panaxcerol B	Lipids	559.311	M+FA-H	C_27_H_46_O_9_	7.31	277.12 559.33 512.66	−	−	−	−	+	−	+	−
43	1-Hexadecanoyl-sn-glycero-3-phospho-(1′-myo-inositol)	Lipid	571.288	[M − H]^−^	C_25_H_49_O_12_P	9.13	153.00 255.23 571.29	−	−	+	+	−	−	−	+
44	Eleutheroside e1	Lignans	579.208	[M − H]^−^	C_28_H_36_O_13_	5.45	181.05 402.12 417.15	−	−	−	−	+	−	−	−
45	Kaempferol 7-neohesperidoside	Flavonoids	593.15	[M − H]^−^	C_27_H_30_O_15_	5.52	284.03 593.15	−	−	+	−	−	−	−	−
46	Kaempferol-3-O-rutinoside	Flavonoids	593.151	[M − H]^−^	C_27_H_30_O_15_	5.52	593.15 594.15	−	−	+	−	+	−	−	−
47	Vicenin-2	Flavonoids	593.151	[M − H]^−^	C_27_H_30_O_15_	4.69	353.06 383.07 473.10 503.11 593.14	−	−	+	−	+	−	−	−
48	6,8-Di-C-glucopyranosylnaringenin	Flavonoids	595.166	[M − H]^−^	C_27_H_32_O_15_	4.27	313.07 355.08 385.09 475.12 595.16	+	−	+	−	−	−	−	−
49	Rutin	Flavonoids	609.147	[M − H]^−^	C_27_H_30_O_16_	5.27	609.13	−	−	+	−	+	−	−	−
50	Compound NP-000390	Flavonoids	623.161	[M − H]^−^	C_28_H_32_O_16_	5.62	314.04 315.05	−	−	−	−	+	−	−	−
51	Isorhamnetin-3-O-rutinoside	Flavonoids	623.204	[M + H]^−^	C_28_H_32_O_16_	5.63	315.06 623.17	−	−	+	−	−	−	−	−
52	Tataramide B	Lignans	623.239	[M − H]^−^	C_36_H_36_N_2_O_8_	6.16	460.18 623.24	−	−	−	+	+	+	−	+
53	Isorhamnetin-3-glucoside-4′-glucoside (Isorhamnetin 3,4′-diglucoside)	Flavonoids	639.156	[M − H]^−^	C_28_H_32_O_17_	4.79	315.05 639.18	−	−	−	−	+	−	−	−
54	(10E,15E)-9,12,13-trihydroxyoctadeca-10,15-dienoic acid	Fatty acids	677.423	2M-2H+Na	C_18_H_32_O_5_	6.53	171.10 183.14 233.12 327.22	−	+	+	+	+	+	+	+
55	(1R,2S)-7-hydroxy-1-(4-hydroxy-3,5-dimethoxyphenyl)-2-N,3-N-bis[2-(4-hydroxyphenyl)ethyl]-6,8-dimethoxy-1,2-dihydronaphthalene-2,3-dicarboxamide	Lignans	683.26	[M − H]^−^	C_38_H_40_N_2_O_10_	5.83	352.12 520.20 683.26	−	−	+	−	+	−	−	−
56	Glc-glc-octadecatrienoyl-sn-glycerol	Lipids	721.364	[M − H]^−^		8.35	235.08 277.22 397.13	−	−	+	−	+	+	+	−
57	Kaempferol 3-O-(2,6-di-O-.alpha.-L-rhamnopyranosyl)-.beta.- D-galactopyranoside	Flavonoids	739.209	[M − H]^−^	C_33_H_40_O_19_	5.18	284.03 285.04	−	−	+	−	−	−	−	−
58	Manghaslin	Flavonoids	755.203	[M − H]^−^	C_33_H_40_O_20_	5.01	300.02 301.03	−	−	+	−	−	−	−	−
59	Kaempferol 3-rutinoside-4′-glucoside	Flavonoids	755.203	[M − H]^−^	C_33_H_40_O_20_	5.01	285.04 447.09 593.15	−	−	+	−	−	−	−	−

+ Present; − Absent. RT: retention time.

**Table 4:** Putatively annotated metabolites detected in *Argemone mexicana* extracts.

S/NO.	Metabolite	Class	Precursor *m*/*z*	Adduct	Molecular Formula	RT (min)	Fragment Ions	Water		Methanol		Acetone		Dichloromethane	
								Shoot	Root	Shoot	Root	Shoot	Root	Shoot	Root
1	Apigenin	Flavonoids	269.045	[M − H]^−^	C_15_H_10_O_5_	4.04	117.03 269.04	−	−	+	−	−	−	−	−
2	Luteolin	Flavonoids	285.04	[M − H]^−^	C_15_H_10_O_6_	5.93	133.03 151.00 285.04	−	−	+	−	−	−	−	−
3	Eriodictyol	Flavonoids	287.055	[M − H]^−^	C_15_H_12_O_6_	5.58	135.04 151.00	−	−	+	−	−	−	−	−
4	6-Gingerol	Phenolic compounds	293.175	[M − H]^−^	C_17_H_26_O_4_	8.66	99.08 293.18	−	−	−	−	−	+	−	+
5	9-hydroxy-10,12-octadecadienoic acid	Fatty acids	295.227	[M − H]^−^	C_18_H_32_O_3_	8.25	171.10 277.22 295.23 296.23	−	−	−	+	−	−	−	+
6	5-hydroxy-6,7-dimethoxyflavone	Flavonoids	297.087	[M − H]^−^	C_17_H_14_O_5_	8.06	183.01 239.03	−	−	−	−	+	+	−	+
7	4-Decylbenzenesulfonic acid	Polyketides	297.152	[M − H]^−^	C_16_H_26_O_3_S	7.98	297.15 298.15	−	−	+	+	+	+	−	+
8	Methyl (2E,4E,8E)-7,13-dihydroxy-4,8,12-trimethyltetradeca-2,4,8-trienoate	Polyketides	309.21	[M − H]^−^	C_18_H_30_O_4_	7.96	183.01	−	−	−	−	+	−	−	−
9	5,6,2′-Trimethoxyflavone	Flavonoids	311.089	[M − H]^−^	C_18_H_16_O_5_	5.24	183.01 311.17	+	+	−	+	+	+	−	+
10	Thymol-beta-D-glucoside	Terpenoids	311.168	[M − H]^−^	C_16_H_24_O_6_	8.40	149.10 183.01 197.03 311.17	+	+	−	+	−	−	+	−
11	Pyrenophorol	Macrolides	311.169	[M − H]^−^	C_16_H_24_O_6_	8.06	183.01 197.03	−	−	+	−	+	+	−	+
12	Feruloyltyramine	Diarylheptanoids	312.123	[M − H]^−^	C_18_H_19_NO_4_	5.48	147.04 148.05 178.05	−	−	+	+	+	+	−	+
13	Isorhamnetin	Flavonoids	315.05	[M − H]^−^	C_16_H_12_O_7_	6.34	151.00 300.02 315.06	−	−	+	−	−	−	−	−
14	Derrustone	Flavonoids	325.073	[M − H]^−^	C_18_H_14_O_6_	3.90	183.01 311.17 325.18	+	+	−	−	−	−	−	+
15	Compound NP-012925	Lignans	325.096	[M − H]^−^	C_19_H_18_O_5_	8.74	183.01 197.03	+	+	+	+	+	+	−	+
16	Compound NP-008285	Macrolides	325.142	[M − H]^−^	C_16_H_22_O_7_	8.75	183.01	+	+	−	+	−	+	−	+
17	4-dodecylbenzenesulfonic acid	Polyketides	325.184	[M − H]^−^	C_18_H_30_O_3_S	8.75	325.18	+	+	+	+	+	+	+	+
18	2,4-dihydroxyheptadec-16-ynyl acetate	Fatty alcohol	325.258	[M − H]^−^	C_19_H_34_O_4_	8.75	79.96 183.01	−	−	−	−	+	+	−	+
19	Docosanol	Fatty acyls	325.346	[M − H]^−^	C_22_H_46_O	8.74	183.01 325.18	−	+	−	−	−	+	−	−
20	9-epi-9-F1t-PhytoP	Fatty acids	327.217	[M − H]^−^	C_18_H_32_O_5_	6.55	171.10 327.22	−	−	−	−	+	−	+	−
21	9,12,13-trihydroxyoctadeca-10,15-dienoic acid	Fatty acids	327.217	[M − H]^−^	C_18_H_32_O_5_	6.54	211.13 229.15 327.22	−	−	−	+	−	+	−	+
22	(-)-scoulerine	Alkaloids	327.574	-	C_19_H_21_NO_4_	6.48	327.22 328.22	−	+	+	+	+	−	+	−
23	9,12,13-trihydroxyoctadec-10-enoic acid	Fatty acids	329.232	[M − H]^−^	C_18_H_34_O_5_	6.71	171.10 211.13 329.23	−	+	+	+	+	+	+	+
24	Compound NP-006769	Terpenoids	339.165		C_18_H_28_O_6_	7.01	183.01	−	−	−	−	−	+	−	−
25	Canrenone	Terpenoids	339.199	[M − H]^−^	C_22_H_28_O_3_	9.10	183.01 33.02	+	+	−	+	+	+	+	+
26	Isocorydine	Alkaloids	341.232	-	C_20_H_23_NO_4_	5.66	341.20 342.20	−	+	+	−	+	+	−	+
27	Angoline	Alkaloids	379.171	-	C_22_H_21_NO_5_	1.32	379.08	−	+	+	−	−	−	+	−
28	(±)-6-Acetonyldihydrochelerythrine	Alkaloids	405.248	-	C_24_H_23_NO_5_	5.44	405.25	−	−	−	−	+	+	+	+
29	Kaempferol-3-O-glucoside	Flavonoids	447.092	[M − H]^−^	C_21_H_20_O_11_	5.49	227.03 255.03 284.03	−	−	+	−	−	−	−	−
30	Kaempferol-3-beta-D-glucopyranoside	Flavonoids	447.092	[M − H]^−^	C_21_H_20_O_11_	5.53	227.03 255.03 284.03 285.04 447.09	−	−	−	−	+	−	−	−
31	Isorhamnetin 3-galactoside	Flavonoids	477.103	[M − H]^−^	C_22_H_22_O_12_	5.62	243.03 271.02 285.04 314.04 477.10	−	−	+	−	+	−	+	−
32	Gossypol	Terpenoids	517.185	[M − H]^−^	C_30_H_30_O_8_	9.05	259.10 471.18 489.19 499.18 517.19	−	+	+	+	+	−	+	+
33	Panaxcerol B	Lipids	559.311	M+FA-H	C_27_H_46_O_9_	7.31	277.12 559.33 512.66	−	−	−	−	+	−	+	−
34	1-Hexadecanoyl-sn-glycero-3-phospho-(1′-myo-inositol)	Lipids	571.288	[M − H]^−^	C_25_H_49_O_12_P	9.13	153.00 255.23 571.29	−	−	+	+	−	−	+	−
35	Eleutheroside e1	Lignans	579.208	[M − H]^−^	C_28_H_36_O_13_	5.45	181.05 402.12 417.15	−	−	+	−	+	−	−	−
36	Kaempferol 7-O-neohesperidoside	Flavonoids	593.15	[M − H]^−^	C_27_H_30_O_15_	5.18	284.03 593.15	−	−	−	−	+	−	−	−
37	Kaempferol-3-O-rutinoside	Flavonoids	593.151	[M − H]^−^	C_27_H_30_O_15_	5.52	593.15 594.15	−	−	+	−	−	−	−	−
38	Vicenin-2	Flavonoids	593.151	[M − H]^−^	C_27_H_30_O_15_	4.69	353.06 383.07 473.10 503.11 593.14	−	−	+	−	−	−	−	−
39	1-(9Z-Octadecenoyl)-sn-glycero-3-phospho-(1′-myo-inositol)	Lipids	597.303	[M − H]^−^	C_27_H_51_O_12_P	9.34	153.00 241.01 281.25 315.07 597.31	−	−	+	−	−	−	−	−
40	Cyanidin-3,5-di-O-glucoside chloride	Anthocyanin chlorides	609.145	[M + H]^−^	C_27_H_31_ClO_16_	5.27	285.04 446.09 447.09	−	−	+	−	−	−	−	−
41	Rutin	Flavonoids	609.147	[M − H]^−^	C_27_H_30_O_16_	5.27	609.13	−	−	+	−	+	−	−	−
42	Compound NP-000390	Flavonoids	623.161	[M − H]^−^	C_28_H_32_O_16_	5.62	314.04 315.05	−	−	+	−	+	−	−	−
43	Isorhamnetin-3-O-galactoside-6″-rhamnoside	Flavonoids	623.161	[M − H]^−^	C_28_H_32_O_16_	5.62	300.02 314.04 315.05 623.16	−	−	+	−	−	−	−	−
44	Isorhamnetin-3-O-rutinoside	Flavonoids	623.204	[M + H]^−^	C_28_H_32_O_16_	5.63	315.06 623.17	−	−	+	−	+	−	−	−
45	Tataramide B	Lignans	623.239	[M − H]^−^	C_36_H_36_N_2_O_8_	6.16	460.18 623.24	−	−	−	+	−	+	−	−
46	Isorhamnetin-3-glucoside-4′-glucoside (Isorhamnetin 3,4′-diglucoside)	Flavonoids	639.156	[M − H]^−^	C_28_H_32_O_17_	4.79	315.05 639.18	−	−	+	−	−	−	−	−
47	Isorhamnetin 3-O-robinoside	Flavonoids	669.166	M+FA-H	C_28_H_32_O_16_	5.60	315.05 623.16	−	−	+	−	+	−	−	−
48	(10E,15E)-9,12,13-trihydroxyoctadeca-10,15-dienoic acid	Fatty acids	677.423	2M-2H+Na	C_18_H_32_O_5_	6.53	171.10 183.14 233.12 327.22	−	+	+	+	+	+	−	−
49	Glc-glc-octadecatrienoyl-sn-glycerol	Lipidss	721.364	[M − H]^−^	C_33_H_56_O_14_	8.35	235.08 277.22 397.13	−	−	+	+	+	−	+	−
50	Kaempferol 3-O-(2,6-di-O-.alpha.-L-rhamnopyranosyl)-.beta.-D-galactopyranoside	Flavonoids	739.209	[M − H]^−^	C_33_H_40_O_19_	5.18	284.03 285.04	−	−	+	−	−	−	−	−
51	Quercetin 3-(2R-apiosylrutinoside)	Flavonoids	741.188	[M − H]^−^	C_32_H_38_O_20_	4.98	255.03 271.02 300.03 741.19	−	−	+	−	−	−	−	−
52	Manghaslin	Flavonoids	755.203	[M − H]^−^	C_33_H_40_O_20_	5.01	300.02 301.03	−	−	+	−	−	−	−	−
53	Compound NP-012184	Flavonoids	771.20	[M − H]^−^	C_33_H_40_O_21_	4.13	301.04 463.09	−	−	+	−	−	−	−	−
54	Isorhamnetin-3-O-glucoside	Flavonoids	955.2	[M + H]^−^	C_22_H_22_O_12_	5.57	477.10	−	−	+	−	−	−	−	−

+ Present; − Absent. RT: retention time.

## Data Availability

The original contributions presented in the study are included in the article; further inquiries can be directed to the corresponding author.

## Abbreviations

The following abbreviations are used in this manuscript:

UPLC-QTOF-MS	Ultra-high-performance liquid chromatography–quadrupole time-of-flight mass spectrometry
PCA	Principal component analysis
OPLS-DA	Orthogonal Partial Least Squares—Discriminant Analysis
Simca	Soft Independent Modeling of Class Analogy
LC-MS	Liquid chromatography–mass spectrometry
AM	*Argemone mexicana*
AO	*Argemone ochroleuca*
AOS	*Argemone ochroleuca* shoots
AOR	*Argemone ochroleuca* roots
AMS	*Argemone mexicana* shoots
AMR	*Argemone mexicana* roots

## References

[1] Bhattacharjee I., Chatterjee S.K., Chandra G. (2010). Isolation and identification of antibacterial components in seed extracts of *Argemone mexicana* L. (Papaveraceae). Asian Pac. J. Trop. Med..

[2] Reyes F.D., Pena C.J., Canales M., Jimenez M., Meraz S., Hernandez T. (2011). Antimicrobial activity of *Argemone ochroleuca* Sweet (Chicalote). Bol. Latinoam. Caribe Plantas Med. Aromat..

[3] Sahu M.C., Debata N.K., Padhy R.N. (2012). Antibacterial activity of *Argemone mexicana* L. against multidrug resistant *Pseudomonas aeruginosa*, isolated from clinical samples. Asian Pac. J. Trop. Biomed..

[4] Reyes-Luna A., Yáñez-Barrientos E., Alba-Mares X.N., Luis Olivares-Romero J., Josabad Alonso-Castro Á., Cruz Cruz D., Villegas Gómez C. (2024). Metabolomic approaches in assessing the insecticidal activity of the extracts from *Argemone ochroleuca* Sweet (Papaveraceae) against three diverse crop pests of economic importance. Chem. Biodivers..

[5] Vardeman E.T., Cheng H.-P., Vandebroek I., Kennelly E.J. (2025). Caribbean medicinal plant *Argemone mexicana* L.: Metabolomic analysis and in vitro effect on the vaginal microbiota. J. Ethnopharmacol..

[6] Singh A., Singh S., Singh S., Singh T., Singh V., Pandey V., Singh U. (2009). Fungal spore germination inhibition by alkaloids dehydrocorydalmine and oxyberberine. J. Plant Prot. Res..

[7] Hernández-Ceja A., Loeza-Lara P.D., Espinosa-García F.J., García-Rodríguez Y.M., Medina-Medrano J.R., Gutiérrez-Hernández G.F., Ceja-Torres L.F. (2021). In vitro antifungal activity of plant extracts on pathogenic fungi of blueberry (*Vaccinium* sp.). Plants.

[8] Kulshrestha S., Goel A., Banerjee S., Sharma R., Khan M.R., Chen K.-T. (2024). Metabolomics and network pharmacology–guided analysis of TNF-α expression by *Argemone mexicana* (Linn) targeting NF-KB the signalling pathway in cancer cell lines. Front. Oncol..

[9] Hernández-Soto I., Juárez-Maldonado A., Madariaga-Navarrete A., Sharma A., Cenobio-Galindo A.D.J., Pinedo-Espinoza J.M., Hernández-Pérez A., Hernández-Fuentes A.D. (2025). Extracts of *Argemone mexicana* L. contain antifungal compounds for the in vitro control of *Monilinia fructicola*, *Colletotrichum gloeosporioides*, *Fusarium oxysporum*, and *Sclerotinia sclerotiorum*: Preliminary evidence for field application. BioTech.

[10] Khan A.M., Bhadauria S. (2019). Analysis of medicinally important phytocompounds from *Argemone mexicana*. J. King Saud Univ. Sci..

[11] Orozco-Nunnelly D.A., Pruet J., Rios-Ibarra C.P., Bocangel Gamarra E.L., Lefeber T., Najdeska T. (2021). Characterizing the cytotoxic effects and several antimicrobial phytocompounds of *Argemone mexicana*. PLoS ONE.

[12] Lokeshwar T., Sathish S. (2023). In vitro phytochemical analysis and antioxidant activity of *Argemone mexicana*. Chelonian Conserv. Biol..

[13] Sharma S., Kumari A., Dhatwalia J., Guleria I., Lal S., Upadhyay N., Kumar V., Kumar A. (2021). Effect of solvents extraction on phytochemical profile and biological activities of Two *Ocimum* Species: A comparative study. J. Appl. Res. Med. Aromat. Plants.

[14] Al-Nemi R., Makki A.A., Sawalha K., Hajjar D., Jaremko M. (2022). Untargeted metabolomic profiling and antioxidant capacities of different solvent crude extracts of *Ephedra foeminea*. Metabolites.

[15] Dunn W.B., Erban A., Weber R.J.M., Creek D.J., Brown M., Breitling R., Hankemeier T., Goodacre R., Neumann S., Kopka J. (2013). Mass Appeal: Metabolite identification in mass spectrometry-focused untargeted metabolomics. Metabolomics.

[16] Sweetlove L.J., Fernie A.R. (2013). The spatial organization of metabolism within the plant cell. Annu. Rev. Plant Biol..

[17] Salem M.A., Perez de Souza L., Serag A., Fernie A.R., Farag M.A., Ezzat S.M., Alseekh S. (2020). Metabolomics in the context of plant natural products research: From sample preparation to metabolite analysis. Metabolites.

[18] Park J., Lee J., Cho C., Lee D.K., Song W., Choi K., Choi S.Y., Yang H. (2025). Mass spectral data of primary and secondary metabolites changes in medicinal plants by solvent polarity. Sci. Data.

[19] Moyo B., Tavengwa N.T., Dubery I., Madala N.E. (2023). Exploration of chemical interactions between *Viscum combreticola* Engl. and its hosts through a metabolic profiling approach and molecular networking. J. Plant Interact..

[20] Waris M., Koçak E., Gonulalan E.M., Demirezer L.O., Kır S., Nemutlu E. (2022). Metabolomics analysis insight into medicinal plant science. Trends Anal. Chem..

[21] Ndou D.L., Ndhlala A.R., Tavengwa N.T., Madala N.E. (2023). A relook into the flavonoid chemical space of *Moringa oleifera* Lam. leaves through a combination of LC-MS and molecular networking. J. Anal. Methods Chem..

[22] Myoli A., Choene M., Kappo A.P., Madala N.E., van der Hooft J.J.J., Tugizimana F. (2024). Charting the cannabis plant chemical space with computational metabolomics. Metabolomics.

[23] Perumal V., Khatib A., Uddin Ahmed Q., Fathamah Uzir B., Abas F., Murugesu S., Zuwairi Saiman M., Primaharinastiti R., El-Seedi H. (2021). Antioxidants profile of *Momordica charantia* fruit extract analyzed using LC-MS-QTOF-based metabolomics. Food Chem. Mol. Sci..

[24] El-Ghorab A.H., Behery F.A., Abdelgawad M.A., Alsohaimi I.H., Musa A., Mostafa E.M., Altaleb H.A., Althobaiti I.O., Hamza M., Elkomy M.H. (2022). LC/MS profiling and gold nanoparticle formulation of major metabolites from *Origanum majorana* as antibacterial and antioxidant potentialities. Plants.

[25] Teoh W.Y., Yong Y.S., Razali F.N., Stephenie S., Dawood Shah M., Tan J.K., Gnanaraj C., Mohd Esa N. (2023). LC-MS/MS and GC-MS analysis for the identification of bioactive metabolites responsible for the antioxidant and antibacterial activities of *Lygodium microphyllum* (Cav.) R. Br. Separations.

[26] Dossou S.S.K., Xu F., Cui X., Sheng C., Zhou R., You J., Tozo K., Wang L. (2021). Comparative metabolomics analysis of different sesame (*Sesamum indicum* L.) tissues reveals a tissue-specific accumulation of metabolites. BMC Plant Biol..

[27] Ntie-Kang F., Lifongo L.L., Mbaze L.M., Ekwelle N., Owono Owono L.C., Megnassan E., Judson P.N., Sippl W., Efange S.M.N. (2013). Cameroonian medicinal plants: A bioactivity versus ethnobotanical survey and chemotaxonomic classification. BMC Complement. Altern. Med..

[28] Lee S., Oh D.-G., Singh D., Lee J.S., Lee S., Lee C.H. (2020). Exploring the metabolomic diversity of plant species across spatial (leaf and stem) components and phylogenic groups. BMC Plant Biol..

[29] Dias M.I., Sousa M.J., Alves R.C., Ferreira I.C.F.R. (2016). Exploring plant tissue culture to improve the production of phenolic compounds: A review. Ind. Crops Prod..

[30] Bjarnholt N., Li B., D’Alvise J., Janfelt C. (2014). Mass spectrometry imaging of plant metabolites—Principles and possibilities. Nat. Prod. Rep..

[31] Motallebi M., Khorsandi K., Sepahy A.A., Chamani E., Hosseinzadeh R. (2020). Effect of rutin as flavonoid compound on photodynamic inactivation against *P. Aeruginosa* and *S. Aureus*. Photodiagnosis Photodyn. Ther..

[32] Miklasińska-Majdanik M., Kępa M., Wąsik T.J., Zapletal-Pudełko K., Klim M., Wojtyczka R.D. (2023). The direction of the antibacterial effect of rutin hydrate and amikacin. Antibiotics.

[33] Yi L., Bai Y., Chen X., Wang W., Zhang C., Shang Z., Zhang Z., Li J., Cao M., Zhu Z. (2024). Synergistic effects and mechanisms of action of rutin with conventional antibiotics against *Escherichia coli*. Int. J. Mol. Sci..

[34] Tarabishi A.A., Mashhoud J., Tahan Z.S. (2024). Quercetin and rutin as a dual approach to antibacterial and anti-biofilm activity via iron chelation mechanism. Discov. Food.

[35] Li A.-P., He Y.-H., Zhang S.-Y., Shi Y.-P. (2022). Antibacterial activity and action mechanism of flavonoids against phytopathogenic bacteria. Pestic. Biochem. Physiol..

[36] Israilov I.A., Yunusov M.S. (1986). Alkaloids of four species of *Argemone*. Chem. Nat. Compd..

[37] Chang Y.-C., Hsieh P.-W., Chang F.-R., Wu R.-R., Liaw C.-C., Lee K.-H., Wu Y.-C. (2003). Two new protopines Argemexicaines A and B and the Anti-HIV alkaloid 6-Acetonyldihydrochelerythrine from formosan *Argemone mexicana*. Planta Med..

[38] Singh A., Singh S., Kesherwani M., Singh T.D., Singh V.P., Pandey V.B., Singh U.P. (2010). Jatrorrhizine and columbamine alkaloids isolated from *Argemone mexicana* are inhibitory to spore germination of some plant pathogenic fungi. Arch. Phytopathol. Plant Prot..

[39] Singh S., Singh A., Jaiswal J., Singh T.D., Singh V.P., Pandey V.B., Tiwari A., Singh U.P. (2010). Antifungal activity of the mixture of quaternary alkaloids isolated from *Argemone mexicana* against some phytopathogenic fungi. Arch. Phytopathol. Plant Prot..

[40] Singh S., Singh A., Keshariwala M., Singh T.D., Singh V.P., Pandey V.B., Singh U.P. (2010). The mixture of tertiary and quaternary alkaloids isolated from *Argemone ochroleuca* inhibits spore germination of some fungi. Arch. Phytopathol. Plant Prot..

[41] Fleck J.D., Betti A.H., Da Silva F.P., Troian E.A., Olivaro C., Ferreira F., Verza S.G. (2019). Saponins from *Quillaja saponaria* and *Quillaja brasiliensis*: Particular chemical characteristics and biological activities. Molecules.

[42] Anuthara R., Jyothis M. (2022). Antibacterial and cytotoxicity studies of gossypol isolated from fruits of *Thespesia populnea* (L.) sol. ex correa- a remedy for skin diseases. Int. J. Adv. Res..

[43] Charalambous D., Christoforou M., Kitiri E.N., Andreou M., Partassides D., Papachrysostomou C., Frantzi M., Karikas G.A., Pantelidou M. (2022). Antimicrobial activities of *Saponaria cypria* Boiss. root extracts, and the identification of nine saponins and six phenolic compounds. Molecules.

[44] Du R.-L., Chow H.-Y., Chen Y.W., Chan P.-H., Daniel R.A., Wong K.-Y. (2023). Gossypol acetate: A natural polyphenol derivative with antimicrobial activities against the essential cell division protein FtsZ. Front. Microbiol..

[45] Moustafa M.F.M., Alamri S.A., Taha T.H., Alrumman S.A. (2013). In Vitro antifungal activity of *Argemone ochroleuca* Sweet latex against some pathogenic fungi. Afr. J. Biotechnol..

[46] Yáñez-Barrientos E., Barragan-Galvez J.C., Hidalgo-Figueroa S., Reyes-Luna A., Gonzalez-Rivera M.L., Cruz Cruz D., Isiordia-Espinoza M.A., Deveze-Álvarez M.A., Villegas Gómez C., Alonso-Castro A.J. (2023). Neuropharmacological effects of the dichloromethane extract from the stems of *Argemone ochroleuca* Sweet (Papaveraceae) and its active compound dihydrosanguinarine. Pharmaceuticals.

[47] Ragunathan V., Pandurangan J., Ramakrishnan T. (2019). Gas chromatography-mass spectrometry analysis of methanol extracts from marine red seaweed *Gracilaria corticata*. Pharmacogn. J..

[48] Ramabulana A.-T., Petras D., Madala N.E., Tugizimana F. (2021). Metabolomics and molecular networking to characterize the chemical space of four *Momordica* plant species. Metabolites.

[49] Makhubu F.N., Siviya L.E., Rauwane M.E., Laurie S.M., Madala N.E., Figlan S. (2025). Biochemical Responses of Atacama and Blesbok Sweet Potato (*Ipomoea batatas* L.) Cultivars to Early Drought Stress. Plants.

[50] Wang M., Carver J.J., Phelan V.V., Sanchez L.M., Garg N., Peng Y., Nguyen D.D., Watrous J., Kapono C.A., Luzzatto-Knaan T. (2016). Sharing and community curation of mass spectrometry data with global natural products social molecular networking. Nat. Biotechnol..

